# Specific Real-Time PCR for the Detection and Absolute Quantitation of Grapevine Roditis Leaf Discoloration-Associated Virus, an EPPO Alert Pathogen

**DOI:** 10.3390/plants9091151

**Published:** 2020-09-04

**Authors:** Félix Morán, Chrysoula-Lito Sassalou, Celia Canales, Varvara I. Maliogka, Antonio Olmos, Ana Belén Ruiz-García

**Affiliations:** 1 Centro de Protección Vegetal y Biotecnología, Instituto Valenciano de Investigaciones Agrarias (IVIA), Ctra. Moncada-Náquera km 4.5, Moncada, 46113 Valencia, Spain; moran_fel@gva.es (F.M.); celia.cq@hotmail.com (C.C.); aolmos@ivia.es (A.O.); 2 Plant Pathology Laboratory, School of Agriculture, Faculty of Agriculture, Forestry and Natural Environment, Aristotle University of Thessaloniki, 54124 Thessaloniki, Greece; sassalou@agro.auth.gr (C.-L.S.); vmaliogk@agro.auth.gr (V.I.M.)

**Keywords:** GRLDaV, plant virus diagnostics, grapevine, real-time PCR

## Abstract

Grapevine Roditis leaf discoloration-associated virus (GRLDaV) is an emerging grapevine pathogen included in the European and Mediterranean Plant Protection Organization (EPPO) alert list due to its ability to damage grapevine crops and cause production losses. This work aimed to develop a specific and reliable diagnostic tool that would contribute to preventing the spread of this pathogen. Therefore, a TaqMan real-time quantitative PCR was developed. The method was validated according to EPPO guidelines showing a high degree of analytical sensitivity, analytical specificity, selectivity, and repeatability and reproducibility. The sensitivity of this method is much higher than the sensitivity reached by previously reported methods even when tested in crude extracts, which could allow rapid testing by avoiding nucleic acid extraction steps. The method was also able to detect GRLDaV isolates from all the geographic origins reported so far, despite their high degree of genetic diversity. In addition, this new technique has been successfully applied for the quantitative detection of GRLDaV in plant material and two mealybug species, *Planococcus citri* and *Pseudococcus viburni*. In conclusion, the methodology developed herein represents a significant contribution to the diagnosis and control of this emerging pathogen in grapevine.

## 1. Introduction

Grapevine (*Vitis vinifera*) is one of the most ancient crops cultivated worldwide for the production of both fresh grapes and wine, which represents an important source of income for many countries. Europe is the leading global wine producer, with about 50% of the world’s vine-growing area, with 3.3 million ha (Food and Agriculture Organization of the United Nations Statistics Division, FAOSTAT, 2018). Grapevine can be affected by virus and virus-like diseases that may significantly reduce the productive life of the plants as well as the grape production in terms of the quantity and quality of the berries [1]. In fact, the grapevine has been described as the crop that hosts the largest number of plant viruses, with more than 85 viral species known to infect this important crop to date [2,3].

Grapevine Roditis leaf discoloration-associated virus (GRLDaV) was identified as a new member of the family *Caulimoviridae*, genus *Badnavirus*, infecting grapevine in 2015 [4]. GRLDaV has been associated with a grapevine disease reported in Greece more than thirty years ago, the Roditis leaf discoloration disease (RLD), which produces discolorations and deformations of the leaves, reduction in the number and size of grape berries, and a lower sugar content [5]. The analysis by high throughput sequencing (HTS) of a grapevine sample showing typical RLD symptoms from a Greek vineyard allowed the identification of GRLDaV and the recovery of its full genome [4]. The GRLDaV genome consists of a single molecule of circular double-stranded DNA (between 6988 and 7097 nt in length) that includes four open reading frames (ORFs) and a non-coding region. ORFs 1, 2, and 4 encode for hypothetical proteins of unknown function. ORF3 encodes a large polyprotein containing the movement protein (MP), the coat protein (CP), the protease, the reverse transcriptase (RT), and the RNaseH [4]. GRLDaV has been shown to be mechanically transmissible. Although no biological vectors have been identified to transmit GRLDaV to date, most badnaviruses are spread by different mealybug species [6].

After its first identification in Greece, GRLDaV has also been reported in Italy [7], Turkey [8], and Croatia [9]. The previous evidence of the ability of this virus to damage grapevine crops and to cause losses, along with its emergence in several European countries, point to the need for the control and prevention of its spread. In fact, GRLDaV was included in the European and Mediterranean Plant Protection Organization (EPPO) alert list as a potential phytosanitary risk for the EPPO region in October 2018. Management and control of viral pathogens and prevention of their spread lay on early detection based on specific and reliable diagnostic methods. Quantitative real-time PCR methods have been shown to be powerful tools for viral epidemiological studies and viral detection [10,11,12,13,14,15]. This work aimed to develop a specific real-time PCR detection method that can perform an absolute quantitation of GRLDaV genetic material in both plant material and mealybugs. Such a technique could be applied to epidemiological studies and would provide a powerful tool to address control strategies designed to prevent GRLDaV spread.

## 2. Results

### 2.1. Detection of GRLDaV by Real-Time qPCR

#### 2.1.1. Design of Real-Time qPCR Primers and Probe

**Figure 1:**
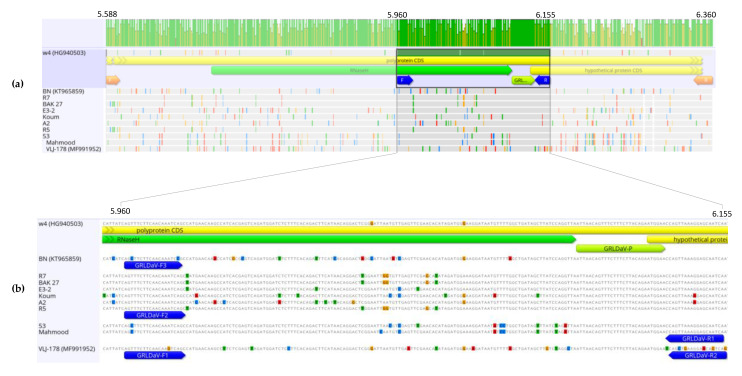
(**a**) Nucleotide alignment of 775 nt partial genomic sequences in the open reading frame 4 (ORF4) region (RNaseH) amplified from Grapevine Roditis leaf discoloration-associated virus (GRLDaV) isolates from different geographical origins. The corresponding region of three GRLDaV full-length sequences available in the databases is included. Alignment was performed by ClustalW implemented in MEGA X software. (**b**) One hundred and ninety-six nucleotide genomic target region, primers (blue arrows), and probe (green arrow) designed for the real-time qPCR developed in this study are indicated.

A nucleotide sequence alignment of the GRLDaV complete genomic sequences available in GenBank, from isolates W4 (Crete, Greece), BN (Italy), and VLJ-178 (Croatia) showed a high degree of divergence between them, with an overall nucleotide identity ranging from 84.24% to 89.31%, although the similarity between the sequences was quite variable throughout the genome. With the aim of selecting a target genomic region that could provide specific, broad, and reliable GRLDaV detection, a more in-depth study was conducted in the RNaseH domain inside the ORF4, one of the most conserved genomic regions used for genus *Badnavirus* specific detection [16]. The sequence variability study in this genomic region led to the selection of a small region of 211 nt (ref-seq genomic positions 5951 to 6161, accession number NC_027131.1), with a nucleotide identity ranging from 85.78% to 93.36% between isolates, as a candidate for primers and probe designing. A larger genomic fragment of 775 nt containing the region of study was amplified by PCR from nine GRLDaV isolates from different geographical origins (Greece and Turkey). The sequences were aligned with the homologous genomic region of the full-length sequences available in the databases from Greece, Italy, and Croatia. (Figure 1).

The sequence alignment showed a highly conserved small region that could be a good candidate for the TaqMan probe. Regions surrounding the alignment site showed some sequence variability that was related to the isolates’ geographical origins. That variability could be overcome by designing either degenerated primers or several primers for use in multiplex PCR. Due to the high number of degenerations to be introduced, the use of degenerated primers could create problems related to primer concentration that could compromise detection sensitivity, as degenerations reduce the final concentration of each particular sequence. Taking this into account, the multiplexing approach was selected.

Thus, five primers able to cover all the variability observed in the sequence alignment and specifically amplify a region of 196 nt (ref-seq genomic positions 5960 to 6155, accession number NC_027131.1) from all isolates having different geographic origins were designed (Figure 1). The primers GRLDaV-F1 (5′AGTTTCTTCAACAAGTCAGC3′, sense), GRLDaV-F2 (5′AGTTTCTTCAACAAATCAGC3′, sense), and GRLDaV-F3 (5′AGCTTCTTCAACAAATCCG3′, sense) exhibited perfect alignment to Croatian, Turkish, and Italian/Greek isolates, respectively. The primer GRLDaV-R1 (5′TGATTGCTCRTTTAACTGG 3′, antisense) included only one degenerated position and was able to align perfectly to all isolates except the Croatian isolate. And the primer GRLDaV-R2 (5′ GACTGTTCCTTCAGCTG 3′, antisense) was designed specifically for the Croatian isolate. Furthermore, a single TaqMan probe specific for all the sequences analyzed was found, GRLDaV-P (5′-6-FAM-ATTAACAGTTTCTTTCTTACAGAATGGAA-BHQ1-ZNA4-3′).

#### 2.1.2. Specific Detection of GRLDaV by Real-Time qPCR

The ability of the real-time qPCR developed in this study to detect GRLDaV was evaluated in 22 isolates from different geographical origins, 17 isolates from Greece (G47 3PR, R5, R7, R8, R11, R12, R14, BAK-27, Koum, D2-1, HL-1, G4-1, G14-2, E3-2, P3, P4, and Auth-48), 2 isolates from Turkey (53 and Mahmood), 2 isolates from Italy (BN GRLDaV 3005 and BN GRLDaV 1405), and 1 isolate from Croatia (VLJ-178). Three replicates of all samples gave a positive detection for GRLDaV, with mean Ct values and their standard deviations ranging from 17.24 ± 0.39 to 32.90 ± 0.42. No signal was detected for negative controls, three tip-culture sanitized grapevine plants and eight GRLDaV-free grapevine plants infected with several common grapevine viruses and viroids (grapevine fanleaf virus, grapevine fleck virus, grapevine leafroll-associated virus 1, grapevine leafroll-associated virus 2, grapevine leafroll-associated virus 3, grapevine red globe virus, grapevine rupestris vein feathering virus, grapevine Syrah virus 1, grapevine virus A, grapevine virus B, grapevine virus E, grapevine virus F, grapevine virus G, grapevine virus L, grapevine yellow speckle viroid-1, grapevine yellow speckle viroid-2, hop stunt viroid and grapevine rupestris stem pitting-associated virus 1).

An additional 65 grapevine plants randomly collected from two Greek grapevine growing areas (Athens and Pieria) were tested by the real-time qPCR method developed. A total of 15 plants were positive for GRLDaV (23.07%) with Ct values ranging from 17.62 to 27.19.

### 2.2. Validation of the Real-Time qPCR Method

#### 2.2.1. Absolute Quantitation and Analytical Sensitivity

**Figure 2:**
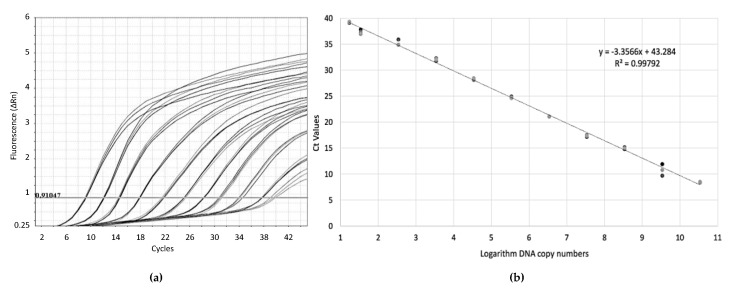
(**a**) Amplification plots by GRLDaV real-time qPCR for three replicates of serial dilutions of plasmid DNA containing the GRLDaV target region. (**b**) Quantitation standard curve generated. Both the mathematical equation of the curve and the coefficient of correlation (R^2^) are indicated.

For the absolute quantitation of the technique, known quantities of the GRLDaV genomic region targeted by the real-time qPCR inserted in a plasmid were used. Serial dilutions of plasmid DNA allowed the generation of standard curves (Figure 2) with an amplification efficiency of 98.57% and a coefficient of correlation of 0.99. The quantitation range of the real-time qPCR was established from 3 × 10^10^ to 20 target copies.

The sensitivity of the developed real-time qPCR in plant material was evaluated using both crude extracts and purified DNA from GRLDaV infected samples. In addition, a sensitivity comparison between our method and two previously reported detection methods, a conventional genus-specific PCR [16], and a GRLDaV specific real-time PCR based on SYBR Green chemistry [4] was carried out. These results are shown in Table 1. The detection limit determined in plant material was around 30 viral copies when testing purified DNA. Crude extracts samples provided a much higher detection limit; a minimal number of 4400 viral targets were needed for detection. The real-time qPCR developed in this study was much more sensitive than the previously described techniques, five orders of magnitude higher than the conventional PCR, and two orders of magnitude higher than the SYBR Green real-time PCR. It is noteworthy that even using crude extracts, the method here developed exhibited higher sensitivity than the currently available diagnostic tools which use purified DNA.

#### 2.2.2. Analytical Specificity and Selectivity

The inclusivity of the technique was evaluated by testing GRLDaV infected plants from different geographic origins showing genetic diversity among them. All isolates tested, representing the high variability observed in this virus, tested positive by the developed method.

The selectivity of the method was also addressed by testing isolates infecting different grapevine cultivars. The results showed that those variations in the matrix did not affect the test performance.

The exclusivity of the real-time PCR was evaluated by analyzing eight grapevine samples infected with several common grapevine viruses (grapevine fanleaf virus, grapevine fleck virus, grapevine leafroll-associated virus 1, grapevine leafroll-associated virus 2, grapevine leafroll-associated virus 3, grapevine red globe virus, grapevine rupestris vein feathering virus, grapevine Syrah virus 1, grapevine virus A, grapevine virus B, grapevine virus E, grapevine virus F, grapevine virus G, grapevine virus L, grapevine rupestris stem pitting-associated virus, grapevine yellow speckle viroid-1, grapevine yellow speckle viroid-2 and hop stunt viroid). All sample viromes were previously determined by HTS. None of these samples tested positive by the GRLDaV real-time qPCR developed in this study (Table 2).

#### 2.2.3. Repeatability and Reproducibility

Repeatability and reproducibility of the real-time qPCR were confirmed by testing seven GRLDaV positive samples with relatively low viral titers in nine technical repeats performed on different days by different operators and using three different thermal cyclers. All assays included three replicates for each sample. Mean Ct values, standard deviations, and coefficient of variation obtained for the technical repeats are shown in Table 3. Mean Ct values and standard deviations ranged from 22.04 ± 1.9 to 35.81 ± 0.6 (crude extract) and from 19.82 ± 0.73 to 33.21 ± 1.33 (purified DNA). The coefficient of variation observed was lower than 9% and ranged from 0.78 to 8.65 (crude extract) and from 1.14 to 8.99 (purified DNA).

### 2.3. Quantitative Detection of GRLDaV in Mealybugs

The real-time qPCR method here developed was applied for the quantitative detection of GRLDaV in two mealybugs species, *Planococcus citri* and *Pseudococcus viburni*. Nine replicates of pools of 2 and 4, as well as individual mealybugs, fed on GRLDaV infected fresh leaves, were analyzed. GRLDaV was successfully detected in all samples, including single mealybugs. No signal was detected for insects fed on healthy grapevine leaves. Viral titers quantified, as well as the mean viral titers for both pools and singles mealybugs and standard deviations, are shown in Supplementary Table S1. Under our experimental conditions, the viral titer determined in *P. citri* in single mealybugs ranged from 99 to 668 viral copies, with a mean viral titer of 321.76 ± 201.97. Viral quantification in mealybugs pools ranged from 128 to 1300 copies (for 2 insect pools) and from 234 to 1325 copies (for 4 insect pools), with a mean viral titer of 651.11 ± 345.59 and 987.33 ± 233.93 copies, respectively (325.55 ± 172.79 and 246.83 ± 108.91 copies per mealybug). For *P. viburni* the mean viral titer in single mealybugs was 136.53 ± 45.61 (from 54 to 192). Quantification on insect pools ranged from 271 to 691 (2 insect pools) and from 179 to 659 (4 insect pools), which represents a mean viral titer of 466.66 ± 155.49 and 506.11 ± 194.10, respectively (233.22 ± 77.74 and 126.53 ± 48.52 per mealybug). GRLDaV acquisition and detection experiments with *P. citri* and *P. viburni* showed the ability of these two mealybug species to carry GRLDaV, and the performance of the real-time qPCR developed in this study to detect and quantify GRLDaV in putative mealybugs.

## 3. Discussion

Quantitative real-time PCR detection methods are powerful and reliable diagnostic tools for the control and management of viral diseases. In this study, we report the development of a specific real-time qPCR method that allows the detection and absolute quantitation of GRLDaV, a grapevine virus included in the EPPO alert list as a potential phytosanitary risk for the EPPO region. This technique performs specific and reliable quantitative detection of GRLDaV in both plant material and mealybugs, a key step to preventing the spread of this pathogen in the European and Mediterranean vineyards.

The GRLDaV genomic sequence shows a high degree of variability, related to its geographical distribution, which might compromise its reliable diagnosis. With the aim of designing a detection method able to deal with this variability, viral sequences from all known GRLDaV-affected geographic locations were analyzed. The real-time qPCR method developed in this study has been shown to specifically cover all known GRLDaV diversity by using a unique TaqMan probe in a highly conserved region of the RNase H domain in ORF4. Five primers, three forward primers, and two reverse primers, working in a single multiplex reaction, were designed to amplify a small region containing the probe target sequence as an alternative of highly degenerated primers that could compromise efficient primer concentrations and detection sensitivity. In fact, the technique has been shown to exhibit not only a high degree of specificity towards its target but also a high level of sensitivity in GRLDaV detection. In addition, this method can also be successfully applied for the absolute quantitation of GRLDaV in both plant material and mealybugs.

The GRLDaV detection method here described has been validated evaluating its analytical sensitivity, analytical specificity, selectivity, and repeatability and reproducibility according to the EPPO guidelines.

First, the method showed a high sensitivity in GRLDaV detection using both crude extracts and purified DNA. In fact, the sensitivity of the technique was much higher than the sensitivity reached by two previously reported detection methods based on conventional generic PCR [16] and SYBR Green real-time PCR [4]. The comparison between the three techniques showed that the sensitivity of the new method was two orders of magnitude higher with respect to the conventional PCR and five orders of magnitude higher with respect to the SYBR Green real-time PCR, with a detection limit of around 30 copies in naturally infected plant material. Moreover, the real-time qPCR developed in this work was able to test crude extracts with a similar sensitivity to the previously reported real-time method testing purified DNA samples. This result raises the possibility of avoiding nucleic acid extraction steps for rapid testing, although sensitivity is two orders of magnitude higher when purified DNA is tested. In any case, the method reported herein provides increased sensitivity in GRLDaV detection compared to existing diagnostic tools.

In addition, the specificity and selectivity of our method were also validated. The assay turned out to be highly specific according to the results obtained for both inclusivity and exclusivity. All GRLDaV isolates analyzed from different geographic origins covering the actual known viral diversity tested positive by the technique. On the other hand, none of the sanitized grapevine nor GRLDaV-free grapevine plants bearing several grapevine common viruses tested positive in our assay, demonstrating the high level of analytical specificity of the method provided herein. In addition, the selectivity of the assay was also shown by its accurate performance in different cultivars of the host plant.

Finally, the repeatability and reproducibility of the method were also demonstrated. A series of nine technical repeats of seven relatively low titer samples tested with three different thermal cyclers on different days and by different operators was successfully performed. The coefficient of variation observed in the analysis results was lower than 9% for all the samples tested, indicating the absence of significant deviations on the technique performance.

The validated technique was applied for the detection of GRLDaV in mealybugs *P. citri* and *P. viburni*. Under our experimental conditions, both mealybug species were able to carry the virus, with their viral titer being successfully quantified by our method. Whether these mealybug species are indeed transmission vectors of this virus remains to be studied. However, the ability of this technique to be applied for those epidemiological studies and used for vector-mediated spread control has been demonstrated.

In conclusion, in this study, we have developed a novel real-time qPCR method for the detection and absolute quantitation of GRLDaV that has been successfully applied for diagnosis in both grapevine plants and mealybugs. The technique has been validated according to EPPO guidelines showing a high sensitivity, specificity, selectivity, and repeatability and reproducibility. This new detection method could contribute to future epidemiological studies and to preventing the spread of this potential pathogen between grapevine plants.

## 4. Materials and Methods

### 4.1. Plant Material

A total of 22 GRLDaV infected grapevine samples from different origins and varieties were selected as positive controls for this study: 17 samples from Greece (cv. Roditis, Dafnia, Mavrothiriko, Moschato Samou, Korinthiaki stafida, Romeiko, Lagorthi, Assyrtiko, and Koumariano); 2 samples from Turkey (cv. Yalova incisi) kindly supplied by Dr. Ulubaş Serçe (Niğde Ömer Halisdemir University); 2 samples from Italy (cv. Bombino Nero) kindly supplied by Dr. Minafra (CNR-IPSP); and 1 sample from Croatia (cv. Ljutun) kindly supplied by Dr. Vončina (University of Zagreb). The presence of GRLDaV in all positive samples was confirmed by PCR using two different sets of primers. Badna-R-Up (5′GAAGGAATTGAATCTCCAGCAGCAGG3′, sense) and Badna-R-Do (5′CTCTGCTACACCAAGTGATAGATTGTTGAG3′, antisense) [4] and Badna-FP (5′ATGCCITTYGGIAARAAYGCICC3′, sense) and Badna-RP (5′CCAYTTRCAIACISCICCCCAICC3′, antisense) [16]. Plant material from grapevines (cv. Bobal, Tempranillo, Marsaoui, Razzegui, Red Globe, and Crimson seedless) analyzed by high throughput sequencing (HTS), harboring different non targeted viruses, and from Spanish grapevine cultivars (cv. Aledo and Ideal) sanitized by tip culture was used as negative control and for specificity assays. In addition, 65 grapevine samples randomly collected from two Greek grapevine growing areas (Athens and Pieria) were analyzed.

### 4.2. Crude Extract Preparation and DNA Purification

Leaf tissue from each plant sample was placed in individual plastic bags (Bioreba, Reinach, Switzerland). Extraction buffer (PBS containing 0.2% diethyldithiocarbamate and 2% PVP-10) was added up to a 1:5 ratio (w:v). Homex 6 homogenizer (Bioreba, Reinach, Switzerland) was used for grinding. For crude extract qPCR real-time analysis, 1:50 plant extract dilutions were performed in H_2_O, centrifuged at 20,000× *g* for 1 min, heated at 95 °C for 10 min, and placed on ice. DNA was purified from 200 µL of the same plant extracts using a Plant/Fungi DNA isolation kit (Norgen Biotek Corporation, Thorold, ON, Canada) following the manufacturer’s instructions. Extracted DNA was quantified with a DeNovix DS-11 spectrophotometer (DeNovix Inc., Wilmington, DE, USA) to determine the DNA concentrations and stored at −80 °C until subsequent analysis.

Total DNA was extracted from mealybugs using a Plant/Fungi Total DNA Purification Kit (Norgen Biotek Corporation, Thorold, ON, Canada) with slight modifications. Briefly, 400 μL of PBS 1X buffer was added to tubes containing 1, 2, 4, or 5 mealybugs. The suspension was vortexed for 5 min in the presence of 212–300 μm glass beads (Sigma-Aldrich, St. Louis, MO, USA). After centrifugation at 10,000× *g* for 5 min, 200 µL of the clarified supernatant was transferred to a fresh tube, and the extraction procedure was completed following the manufacturer’s instructions.

### 4.3. Amplification and Sequencing of a Partial GRLDaV RNaseH/ORF4 Genomic Region

Alignment of the three GRLDaV full genomes available in the databases [17] corresponding to isolates W4, BN, and VLJ-178 from Greece, Italy, and Croatia, respectively (accession numbers HG940503, KT965859, and MF991952) was performed using ClustalW implemented in Geneious software 10.0.7 (Biomatters Ltd., Auckland, New Zealand). Based on this alignment, primers targeting a 775 nt in the GRLDaV genome including part of the RNaseH sequence (ORF3) and part of the ORF4 region, GRLDaV-RNaseH-F (5′AGCCCACTCTATGCYAARACAAG3′, sense), and GRLDaV-RNaseH-R (5′TGCTTTCTTTGCTGACTCAGCTTC3′, antisense) were designed manually. Primers and probe melting temperatures and secondary structures were analyzed using the online OligoAnalyzer Tool (Integrated DNA Technologies Inc., Coralville, IA, USA). A 775 bp fragment was amplified from nine selected GRLDaV positive samples from different origins: sample R5 (Crete, Greece); sample R7 (Crete, Greece); sample BAK-27 (Nemea, Peloponese, Greece); sample Koum (Tinos island, Greece); sample D2-1 (Grapevine Institute, Athens, Greece); sample E3-2 (Grapevine Institute, Athens, Greece); sample VLJ-178 (Kaštela, Croatia); sample 53 (Adana province, Turkey) and sample Mahmood (Adana Province, Turkey). PCR amplification was performed using Platinum Taq DNA polymerase (Thermo Fisher Scientific, Waltham, MA, USA) following the manufacturer’s instructions. The reaction mixture contained 0.5 µM of each primer, 0.75 µL of KB Extender, and 40 ng of DNA in a volume of 3 µL, in a total reaction volume of 25 µL. PCR protocol consisted of a first step at 94 °C for 2 min, followed by 35 cycles of amplification (30 s at 94 °C, 30 s at 55 °C, and 48 s at 72 °C). All PCR amplicons were cleaned using mi-PCR Purification Kit (Metabion International AG, Martinsried, Germany), and Sanger sequenced in both directions.

### 4.4. Primers and Probe Design

All amplified RNaseH/ORF4 sequences, as well as the corresponding genomic region of the GRLDaV full-length sequences available in the databases (HG940503, KT965859, and MF991952), were aligned using ClustalW implemented in MEGA X software [18]. Based on the alignment, a set of five primers and a probe were designed for the real-time PCR assay. Three forward primers and two reverse primers were designed to cover all the variability detected in the selected region, yielding an amplicon of 196 bp: GRLDaV-F1 (5′AGTTTCTTCAACAAGTCAGC3′, sense); GRLDaV-F2 (5′AGTTTCTTCAACAAATCAGC3′, sense); GRLDaV-F3 (5′AGCTTCTTCAACAAATCCG3′, sense); GRLDaV-R1 (5′TGATTGCTCRTTTAACTGG 3′, antisense); and GRLDaV-R2 (5′ GACTGTTCCTTCAGCTG 3′, antisense). A TaqMan probe, GRLDaV-P (5′-6-FAM-ATTAACAGTTTCTTTCTTACAGAATGGAA-BHQ1-ZNA4-3′) showed specificity towards all the sequences used in the study.

### 4.5. TaqMan Quantitative Real-Time PCR

TaqMan qPCR was performed in three different real-time thermal cyclers: StepOne Plus thermal cycler (Applied Biosystems, Foster City, CA, USA); QuantStudio 3 real-time PCR system (Applied Biosystems, Foster City, CA, USA), and LightCycler 480 (Roche, Basel, Switzerland), using a Premix Ex Taq master mix for probe-based real-time PCR (Takara Bio Inc., Kusatsu, Japan) according to the manufacturer’s instructions. Five microliters containing 70 ng of DNA or 5 µL of crude extract were used as a template. The reaction mixture contained 250 nM of the probe and 0.35 µM of each of the primers in a total reaction volume of 20 µL. For reactions performed in the Applied Biosystems instruments, 0.4 µL of the ROX Reference Dye (50X) was added as a passive reference dye. PCR reaction consisted of an initial denaturation step at 95 °C for 2 min, followed by 45 cycles of amplification (15 s at 95 °C and 1 min at 60 °C). Data acquisition and analysis were performed using the corresponding software for each thermal cycler. The default threshold set by the machines was slightly adjusted above the noise to the linear part of the growth curve at its narrowest point, according to the manufacturer.

### 4.6. Generation of Real-Time qPCR Standard Curves

For the generation of real-time qPCR standard curves, the RNaseH fragment targeted by the qPCR was amplified by conventional PCR using primers GRLDaV-F2 and GRLDaV-R1. The 196 bp PCR product was purified using a PCR Purification Kit (Metabion International AG, Martinsried, Germany) following the manufacturer’s instructions, inserted in the pGEM-T Easy vector (Promega Corporation, Madison, OH, USA), and cloned into *Escherichia coli* XL1-Blue. Transformant colonies were selected by ampicillin resistance. Plasmid extraction was performed using the mi-Plasmid Miniprep Kit (Metabion International AG, Martinsried, Germany) following the manufacturer’s instructions and quantified with a DeNovix DS-11 spectrophotometer (DeNovix Inc., Wilmington, DE, USA). Plasmid DNA was quantified in picomoles applying the following mathematical formula: pmol of dsDNA = (μg of dsDNA × 10^6^)/(660 × Nbp) considering the average molecular weight of a pair of nucleotides (660 Da) and the number of base pairs of the plasmid carrying the GRLDaV partial genomic RNaseH sequence (Nbp). The Avogadro constant [19] was used to estimate the number of plasmid molecules (6.023 × 10^23^ molecules/mol). Three replicates of serial dilutions from 3 × 10^10^ to 2 plasmid copies were prepared and used to generate the standard curve. The amplification efficiency of the calibration curve was calculated by the slope, according to the mathematical formula: amplification efficiency = [10 ^(−1/slope)^]−1 [20].

### 4.7. Validation of the Real-Time qPCR Method

The real-time qPCR method developed in this study was validated according to the EPPO guidelines [21]. Thus, analytical sensitivity (maximum dilution of DNA detected), analytical specificity (inclusivity, detection of variants/strains covering genetic diversity and different geographic origin; exclusivity, negative detection of relevant non-targets that might be present in the matrix), selectivity (evaluation of test performance using different cultivars), and repeatability and reproducibility (evaluation of the test performance consistency on different replicates by different operators and with different equipment) were evaluated.

#### 4.7.1. Evaluation of the Sensitivity

Analytical sensitivity of the real-time PCR developed in this study was evaluated using crude extracts or purified DNA from GRLDaV naturally infected plant material. The crude extracts were tested at 1:50 dilution in distilled H_2_O. DNA was purified from GRLDaV infected plant extract and from ten-fold serial dilutions (from 10^−1^ to 10^−8^) of the infected plant extract in healthy plant extract, to avoid dilution of putative PCR inhibitors present in the matrix. Absolute quantitation was performed, generating standards curves as described above, and viral titer, the number of viral targets per volume, was determined. Three replicates of each sample were tested. All samples were tested by two previously described GRLDaV detection methods [4,16] for sensitivity comparison.

#### 4.7.2. Evaluation of the Specificity

The inclusivity and selectivity of the developed detection method were evaluated by testing GRLDaV isolates from different geographic origins showing genetic diversity and infecting different cultivars.

Exclusivity was evaluated by testing eight grapevine samples infected by several viruses commonly present in the host. The virome of these samples, not infected by GRLDaV, was determined by high throughput sequencing (HTS), as described previously [22]. Briefly, total RNA was extracted from leaves and sequenced after ribo-depletion using RNAseq TrueSeq Illumina technology (150 nt pair-end reads). Data analysis was performed by CLC Genomics Workbench 10.1.1 (QIAGEN, Hilden, Germany). Raw data were subjected to quality control, trimming, and host genome subtraction steps, and the resulting reads used to assemble de novo contigs (minimal contig length 200 nt). The contigs similarity to viral sequences was analyzed by BLASTn and BLASTx with a cut-off e-value of 10^−4^.

#### 4.7.3. Evaluation of the Repeatability and Reproducibility

For evaluation of the repeatability and reproducibility of the real-time qPCR, seven surveyed samples that tested positive for GRLDaV with the lowest viral titers were selected. Both crude extract and purified DNA of the selected samples were tested in nine technical repeats using three different thermal cyclers, QuantStudio 3 real-time PCR system Plus (Applied Biosystems, Foster City, CA, USA), StepOne Plus (Applied Biosystems, Foster City, CA, USA), and LightCycler 480 (Roche). For each of the instruments, 3 independent repeats were performed, carried out on different days, and by different operators. Technical repeats included 3 replicates for each sample. Mean Ct values and coefficients of variation were calculated.

### 4.8. Quantitative Detection of GRLDaV in Mealybugs

Colonies of the *P. citri* and *P. viburni* mealybugs were kindly provided by Dr. Beitia (IVIA). Adult mealybugs were originally collected from persimmon fruits (*Diospyros kaki* L.) located in a persimmon growing area in Algemesí (Ribera del Xúquer, Valencia, Spain). Mealybug colonies were maintained in lemon fruits at 26 °C for 60 days. During this period, the absence of GRLDaV in the mealybugs was confirmed twice, every 30 days, by testing 5 pools of 5 mealybugs by both the conventional and the real-time GRLDaV PCR detection methods previously described [4,16] and by the real-time qPCR method developed in this study. GRLDaV-free mealybugs were transferred to both GRLDaV infected and healthy grapevine leaves. Acquisition was performed at 28 °C, 60% humidity and a 12/12 h light cycle for 48 h in a plant growth chamber (Sanyo MLR-350H). After this time, pools of 2 and 4 mealybugs, as well as individual insects, were analyzed by the real-time qPCR developed in this study. These experiments were performed in nine technical repeats.

## Figures and Tables

**Table 1:** Analytical sensitivity of the real-time qPCR developed compared to the sensitivity of two previously reported Grapevine Roditis leaf discoloration-associated virus (GRLDaV) detection methods [4,16].

	Real-Time qPCR (This Study)		SYBR Green Real-Time PCR [4]	Conventional PCR [16]
Serial Dilutions ^1^	Viral Titer ^2^ (Purified DNA)	Signal (Crude Extract)	Signal (Purified DNA)	Signal (Purified DNA)
undiluted	5.22 × 10^6^	+	+	+
10^−1^	7.18 × 10^8^	+	+	+
10^−2^	6.31 × 10^7^	+	+	+
10^−3^	1.01 × 10^6^	+	+	+
10^−4^	2.74 × 10^5^	+	+	−
10^−5^	2.70 × 10^4^	+	+	−
10^−6^	4.40 × 10^3^	+	+	−
10^−7^	3.30 × 10^2^	−	−	−
10^−8^	2.88 × 10^1^	−	−	−

^1^ Ten-fold serial dilutions of GRLDaV infected grapevine extract in a healthy plant extract. ^2^ Mean viral titer (number of viral targets per 5 µL of sample) of three replicates as determined by the real-time qPCR assay designed in this study.

**Table 2:** High throughput sequencing (HTS)-determined virome of eight grapevine samples used to evaluate the exclusivity of the real-time qPCR developed.

Sample Code	Variety	Total Numbers of Reads ^1^	*De Novo* Contigs	Virome ^2^
M2	Marsaoui	686,277	3019	GLRaV-1; GLRaV-2; GLRaV-3; GVF; GVA; GVB; RSPaV-1; GLRaV-2; GFLV; GYSVd-1; HSVd; GVL
U24	Bobal	3,095,597	1943	GLRaV-1; GLRaV-3; GYSVd-1; HSVd
R1	Red Globe	918,235	7884	GLRaV-2; GLRaV-3; GFkV; GVF; GVA; GFLV; GSyV-1; GYSVd-1; GYSVd-2; HSVd; GVL
R35	Bobal	3,735,091	5852	GLRaV-3; GRVFV; GFkV; GYSVd-1; HSVd
R5	Tempranillo	2,702,189	16,961	GLRaV-3; GRVFV; RSPaV-1; GVA; GRGV; GYSVd-1; HSVd
R3	Razzegui	1,647,485	6018	GPGV; GVF; GLRaV-1; GLRaV-2 GLRaV-3; GVA; GFkV; GVG; GVK; GFLV; RSPaV-1; GVB; HSVd; GYSVd-1; GVL
Pin1	Crimson seedless	8,642,008	14,375	GRVFV; GFkV; RSPaV-1; GVA; GLRaV-2; GYSVd-1; HSVd
R4	Tempranillo	2,410,654	13,303	RSPaV-1; GLRaV-3; GVA; GFkV; GRGV; GRVFV; GYSVd-1; HSVd

^1^ After host genome subtraction. ^2^ Grapevine fanleaf virus (GFLV); Grapevine fleck virus (GFkV); Grapevine leafroll-associated virus 1 (GLRaV-1); Grapevine leafroll-associated virus 2 (GLRaV-2); Grapevine leafroll-associated virus 3 (GLRaV-3); Grapevine red globe virus (GRGV); Grapevine rupestris vein feathering virus (GRVFV); Grapevine Syrah virus 1 (GSyV-1); Grapevine virus A (GVA); Grapevine virus B (GVB); Grapevine virus E (GVE); Grapevine virus F (GVF); Grapevine virus G (GVG); Grapevine virus L (GVL); Grapevine yellow speckle viroid-1 (GYSVd-1); Grapevine yellow speckle viroid-2 (GYSVd-2); Hop stunt viroid (HSVd); Grapevine rupestris stem pitting-associated virus 1 (RSPaV-1).

**Table 3:** Repeatability and reproducibility of the real-time qPCR method. Each sample was analyzed in nine technical repeats in three different thermal cyclers (three repeats per instrument), testing both crude extracts and purified DNA.

	Crude Extract						Purified DNA					
	StepOne Plus		QuantStudio		Roche 480		StepOne Plus		QuantStudio		Roche 480	
Sample Code	Mean Ct ± SD	CV (%)	Mean Ct ± SD	CV (%)	Mean Ct ± SD	CV (%)	Mean Ct ± SD	CV (%)	Mean Ct ± SD	CV (%)	Mean Ct ± SD	CV (%)
60.5	28.28 ± 1.42	5.03	30.63 ± 1.12	1.82	33.04 ± 0.29	0.91	22.95 ± 1.11	5.56	26.72 ± 0.63	2.37	27.41 ± 0.94	3.46
60.11	22.04 ± 1.90	8.65	24.74 ± 1.25	5.08	26.04 ± 0.86	3.32	20.94 ± 0.92	8.90	22.48 ± 1.92	8.54	23.09 ± 1.10	4.77
60.15	26.90 ± 1.6	6.22	28.92 ± 0.9	3.17	29.77 ± 0.86	2.91	21.09 ± 0.60	2.87	24.89 ± 0.43	1.62	25.82 ± 0.29	1.14
60.16	25.91 ± 1.16	6.31	27.86 ± 1.5	2.32	31.31 ± 0.45	1.12	20.73 ± 1.70	3.35	23.30 ± 0.49	2.13	23.97 ± 0.70	13.65
60.17	27.78 ± 1.48	5.33	29.21 ± 0.22	0.78	30.36 ± 0.36	1.19	21.15 ± 0.52	5.24	24.55 ± 0.29	1.19	26.04 ± 0.76	1.72
60.21	32.05 ± 2.08	6.51	35.81 ± 0.6	5.89	30.52 ± 0.23	1.16	27.05 ± 0.56	2.07	30 ± 0.42	1.43	33.21 ± 1.33	8.99
60.23	22.95 ± 1.42	6.23	24.70 ± 0.3	1.25	27.12 ± 0.61	2.25	19.82 ± 0.73	4.69	21.98 ± 0.88	4.01	23.76 ± 1.41	5.94

## Supplementary Materials

The following are available online at https://www.mdpi.com/2223-7747/9/9/1151/s1. Table S1: GRLDaV titer in both pools and single mealybugs quantified by the real-time qPCR (species *P. citri* and *P. viburni*).
