# Supplementary material for: Specific Real-Time PCR for the Detection and Absolute Quantitation of Grapevine Roditis Leaf Discoloration-Associated Virus, an EPPO Alert Pathogen
Source: Plants (Basel). 2020 Sep 4;9(9):1151. doi: 10.3390/plants9091151 (PMC7569790; doi:10.3390/plants9091151)
Supplement: Supplementary file 1 [file plants-09-01151-s001.pdf]

Table S1. GRLDaV viral titter in both pools and singles mealybugs quantified by the real-time qPCR (species *P. citri* and *P. viburni*).

Number of insects	Sample code	Viral titter	Viral titter per mealybug	Mean viral titter ± SD	Mean viral titter per mealybug ± SD
**1** ***P. citri***	1.1	545	-	321.76 ± 201.97	-
	1.2	99			
	1.3	284			
	1.4	342			
	1.5	294			
	1.6	346			
	1.7	668			
	1.8	317			
	1.9	0			
**2** ***P. citri***	2.1	1300	650	651.11 ± 345.59	325.55 ± 172.79
	2.2	128	64		
	2.3	756	378		
	2.4	451	225.5		
	2.5	991	495.5		
	2.6	726	363		
	2.7	382	191		
	2.8	581	290.5		
	2.9	545	272.5		
**4** ***P. citri***	4.1	234	78	987.33 ± 233.93	246.83 ± 108.91
	4.2	885	295		
	4.3	1232	410.6		
	4.4	945	315		
	4.5	936	312		
	4.6	923	307.6		
	4.7	1325	441.6		
	4.8	1235	411.6		
	4.9	1168	389.3		
**1** ***P. viburni***	1.1	174	-	136.53 ± 45.61	-
	1.2	138			
	1.3	92			
	1.4	192			
	1.5	54			
	1.6	174			
	1.7	159			
	1.8	146			
	1.9	100			
**2** ***P. viburni***	2.1	295	147.5	466.66 ± 155.49	233.22 ± 77.74
	2.2	498	249		
	2.3	280	140		
	2.4	691	345.5		
	2.5	558	279		
	2.6	428	214		
	2.7	271	135.5		
	2.8	588	294		
	2.9	589	294.5		
**4** ***P. viburni***	4.1	179	44.7	506.11 ± 194.10	126.53 ± 48.52
	4.2	299	74.7		
	4.3	277	69.2		
	4.4	657	164.2		
	4.5	659	164.7		
	4.6	619	154.7		
	4.7	629	157.2		
	4.8	616	154		
	4.9	620	155		
